# Progress in Developing Inhibitors of SARS-CoV-2 3C-Like Protease

**DOI:** 10.3390/microorganisms8081250

**Published:** 2020-08-18

**Authors:** Qingxin Li, CongBao Kang

**Affiliations:** 1 Guangdong Provincial Engineering Laboratory of Biomass High Value Utilization, Institute of Bioengineering, Guangdong Academy of Sciences, Guangzhou 510316, China; 2 Experimental Drug Development Centre (EDDC), Agency for Science, Technology and Research (A*STAR), 10 Biopolis Road, Chromos, #05-01, Singapore 138670, Singapore

**Keywords:** COVID-19, SARS-CoV-2, drug discovery, protease inhibitor, protein structures, antivirals, drug discovery

## Abstract

Coronavirus disease 2019 (COVID-19) is caused by severe acute respiratory syndrome coronavirus 2 (SARS-CoV-2). The viral outbreak started in late 2019 and rapidly became a serious health threat to the global population. COVID-19 was declared a pandemic by the World Health Organization in March 2020. Several therapeutic options have been adopted to prevent the spread of the virus. Although vaccines have been developed, antivirals are still needed to combat the infection of this virus. SARS-CoV-2 is an enveloped virus, and its genome encodes polyproteins that can be processed into structural and nonstructural proteins. Maturation of viral proteins requires cleavages by proteases. Therefore, the main protease (3 chymotrypsin-like protease (3CL^pro^) or M^pro^) encoded by the viral genome is an attractive drug target because it plays an important role in cleaving viral polyproteins into functional proteins. Inhibiting this enzyme is an efficient strategy to block viral replication. Structural studies provide valuable insight into the function of this protease and structural basis for rational inhibitor design. In this review, we describe structural studies on the main protease of SARS-CoV-2. The strategies applied in developing inhibitors of the main protease of SARS-CoV-2 and currently available protein inhibitors are summarized. Due to the availability of high-resolution structures, structure-guided drug design will play an important role in developing antivirals. The availability of high-resolution structures, potent peptidic inhibitors, and diverse compound scaffolds indicate the feasibility of developing potent protease inhibitors as antivirals for COVID-19.

## 1. Introduction

Coronavirus disease 2019 (COVID-19) is caused by severe acute respiratory syndrome coronavirus 2 (SARS-CoV-2), and the viral outbreak started in December 2019 [1,2,3]. The disease spread worldwide, and the World Health Organization (WHO) declared COVID-19 a pandemic in March 2020. According to the WHO, COVID-19 cases have been found in 215 countries, areas, or territories, with over 20 million cases reported and over 700 thousand deaths from this disease as of August 2020. Many countries have taken actions to stop the spread of the virus as it can spread among people through close contact [4]. Strategies such as reducing transportation across different countries were implemented to prevent the spread of the virus, causing a huge amount of revenue loss globally in various fields. The lifestyle of people has been affected due to the pandemic as no specific pharmaceutical treatments are available. It is estimated that some of the interventions, such as social distancing, might extend until 2022 [5]. Therefore, chemotherapies and vaccines are needed to combat and prevent this disease [2]. Progress has been made in drug discovery and development since the outbreak of COVID-19 [6,7,8,9]. For example, remdesivir, an inhibitor of RNA polymerase, has been approved by the Food and Drug Administration (FDA), although more antivirals are still required [10]. Several vaccines and some drug candidates have entered clinical trials, but studies to develop novel antivirals and vaccines are still important to treat this disease due to clinical needs and mutations in the virus that might affect the efficacy of the antivirals [11,12].

**Figure 1:**
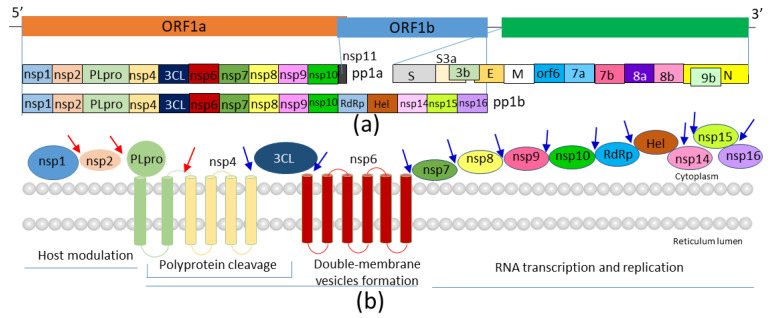
Genome of severe acute respiratory syndrome coronavirus 2 (SARS-CoV-2). (**a**) Viral proteins encoded by the viral genome. The nonstructural proteins (nsps), structural proteins, and accessory proteins (Orf3a to Orf9b) are shown. (**b**) Membrane topology of several nonstructural proteins. The transmembrane domains of proteins are shown as cylinders. Arrows indicate cleavage sites of: papain-like cysteine protease (PL^pro^; red) and 3 chymotrypsin-like protease (3CL^pro^; blue). Other nonstructural proteins are shown as spheres. The sphere has not been drawn to actual scale of individual proteins. More information can be obtained from https://viralzone.expasy.org/764.

Coronaviruses are enveloped viruses that are found in avian and mammalian species [3]. They are classified into four genera: alpha-, beta-, gamma-, and delta-coronaviruses [2]. The beta-coronavirus class comprises important human pathogens, such as SARS-CoV, which caused a viral outbreak in 2003 [13]; Middle East respiratory syndrome (MERS) coronavirus, which occurred in 2012 [14]; and SARS-CoV-2, which is currently a direct threat to global population [1]. SARS-CoV and SARS-CoV-2 are closely related, and these viruses might have originated from bats [15]. Viral genome is a single-strand, positive-sense RNA with a size of ~30 kb, which contains quite a few open-reading frames [15]. Two-thirds of the viral genome encodes 16 nonstructural proteins (nsp1–16), while the remaining genome encodes four structural and nine accessory proteins (Orf3a, Orf3b, Orf6, Orf7a, Orf7b, Orf8, Orf9b, Orf9c, and Orf10) [15]. These viral proteins are critical for the life cycle of viruses as they participate in forming viral particles, determining viral replication, and interacting with host proteins to modulate immune response [16]. Viral particles of coronaviruses contain four main structural proteins: spike (S), membrane (M), envelope (E), and nucleocapsid (N) proteins. The viral genome is encapsulated within a membrane envelope, where glycoprotein spikes make coronaviruses appear crown-like. The transmembrane S protein is a glycoprotein and forms homotrimers on the viral surface [17]. S is an important target because it is critical for viruses entering human cells by interacting with angiotensin-converting enzyme 2 (ACE2) [17,18,19]. Structural studies have elucidated the molecular basis for S and ACE2 interactions, and disrupting this interaction could be one strategy to prevent viral infection [20]. The M protein is a small membrane protein with three transmembrane domains, which might be important for the virion shape. The E protein is a transmembrane protein that is important for pathogenesis [21]. The N protein plays an important role in RNA package and release of viral particles. Several nonstructural proteins harbor enzymatic activities, such as protease and RNA-directed RNA polymerase activities. Blocking these enzymatic activities will be an effective strategy for developing antivirals against SARS-CoV-2 (Figure 1). Several reviews have described strategies applied for developing antivirals against COVID-19 [6,22,23,24,25,26,27,28,29,30]. This review provides an overview of efforts made in developing inhibitors of the SARS-CoV-2 protease 3 chymotrypsin-like protease (3CL^Pro^, also called main protease). The structure of SARS-CoV-2 3CL^Pro^, strategies applied for developing protease inhibitors, and the progress in inhibitor development are described.

## 2. Structures of SARS-CoV-2 3CL^Pro^

Similar to other coronaviruses, the genome of SARS-CoV-2 encodes several polyproteins that need to be processed into functional proteins [15]. The two large replicase polyproteins, namely, pp1a (~450 kDa) and pp1ab (~750 kDa), share high sequence homology with those of SARS-CoV [20]. These polyproteins are further processed by viral proteases to produce functional proteins critical for viral replication (Figure 1). The viral genome encodes two proteases: papain-like cysteine protease (PL^pro^) and 3CL^Pro^. PL^pro^ has three cleavage sites in pp1ab to release the first three proteins (Figure 1). 3CL^Pro^ cleaves no less than 11 sites of polyproteins, which is critical for releasing nsp4–nsp16 [31]. PL^pro^ is an important target for developing antivirals as it is a multifunctional protein harboring protease, deISGylating, and deubiquitinating activities [6,32,33]. SARS-CoV-2 PL^pro^ recognizes a sequence motif containing LXGG↓(A/K)X, where X is any type of amino acid [6,34,35]. 3CL^Pro^ is a cysteine protease and recognizes a sequence with Leu and Gln at P2 and P1 positions, respectively. This review is focused on 3CL^Pro^, which recognizes multiple sites of the viral protein. Extensive structural and drug discovery studies have been conducted on 3CL^Pro^, and they have shown that this protease is a validated target for developing antivirals [29] as inhibiting its activity will block maturation of viral proteins that are indispensable for viral replication.

**Figure 2:**
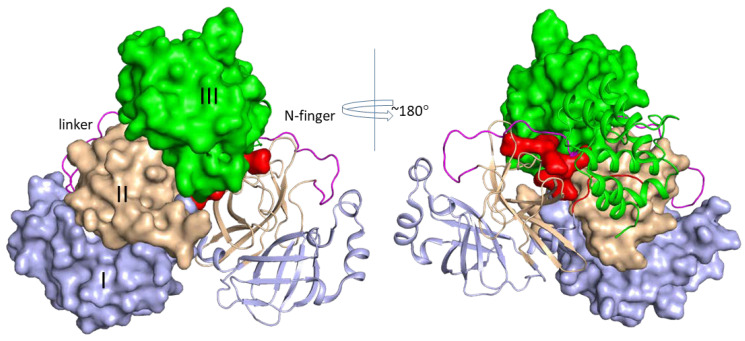
Structure of SARS-CoV-2 3CL^pro^. The N-terminal seven residues (N-finger), domains I, II, III, and the linker of domains II and III of both protomers are shown in red, light blue, wheat, green, and purple, respectively. The linker in the two protomers is shown in ribbon mode. Other domains in one protomer are shown in surface mode except the linker region, and corresponding domains in the other protomer are shown in ribbon mode. The structure (PDB ID 6Y2G) is used in this figure.

Crystal structures of SARS-CoV-2 3CL^pro^ in the absence and presence of inhibitors have been established [31,36], and over 100 structures have been deposited in the protein data bank (PDB) (Table 1). It is not surprising that the folding of SARS-CoV-2 3CL^pro^ is identical to that of SARS-CoV due to their high sequence identity [37]. The crystal structures of 3CL^pro^ show that the protease forms dimers, and two promoters are packed at almost a right angle [36,38,39,40] (Figure 1). The protease contains three domains, namely, domain I (residues 8–101), domain II (residues 102–184), and domain III (residues 201–303) (Figure 2) [36,40]. Domains I and II have an antiparallel β-barrel structure, which is similar to structures of the trypsin-like serine proteases. Domain III consists of five α-helices and is connected with domain II via a long loop composed of residues 185–200. Domain III is not involved in direct interaction with the substrate, but it is critical for enzymatic activity of proteins as removing this domain results in an inactive protease [41]. A catalytic dyad of SARS-CoV 3CL^pro^ is formed by Cys145 and His41, which is different from the Ser–His–Asp catalytic triad in a serine protease [42]. The substrate/inhibitor binds to the cleft, which is located between domains I and II. 3CL^pro^ of coronavirus has a preference for Gln residing in the P1 position. The corresponding S1 site of the protease is composed of side chains of His163 and Phe140 and main-chain atoms of Met165, Glu166, and His172 [40].

SARS proteases have been shown to form dimers in crystallographic studies, and a crystal structure containing octamer SARS-CoV 3CL^pro^ was observed in one study [43]. Purified recombinant protease contain both monomeric and dimeric forms in solution, while the dimeric form of SARS 3CL^pro^ is necessary for the enzymatic activity [44]. Domain II of one molecule interacts with N-terminal seven residues (N-finger) of the other molecule. This interaction is critical for protease dimerization as the N-finger interacting with Glu166 is able to shape the S1 pocket [45]. Mutations in the dimeric interface affect the protein structure [46] or abolish dimer formation [47]. Domain III is also critical for protease dimerization, and NMR studies have shown that domain III does not affect secondary structures of other domains [48]. A polar interaction between two domains III in SARS-CoV exists in crystal structures. A hydrogen bond between side chains of Thr285 from two protomers is present, which is supported by hydrophobic contact between Ile286 and Thr285. The interaction does not exist in SARS-CoV-2 3CL^pro^, in which Thr285 is replaced with an Ala and Ile286 is replaced with a Leu [36]. Mutation in these residues was found to cause enhancement of the catalytic activity of SARS-CoV 3CL^pro^ mutants, which might be due to some changes in the protein dynamics [49]. Mutation in other residues, such as Glu288Ala and Asp289Ala, resulted in proteases with lower activities than the wild type [50]. Solution NMR studies have suggested that N-terminal five residues and Arg298 are critical for dimerization as protease mutants lacking the first five residues or harboring a Arg298/Ala mutation exhibited NMR spectra different from that of the wild type [50]. A study on SARS-CoV 3CL^pro^ showed that recombinant domain III contained both monomers and dimers in solution, while the N-finger was critical for forming the active protease dimer [51]. These results are consistent with a biochemical study that demonstrated the catalytic efficiency of 3CL^pro^ of SARS-CoV-2 was only slightly higher than the protease from SARS-CoV despite of their amino acid difference in domain III [31,36]. These biochemical and structural studies provide valuable information to design potent inhibitors and understand the regulation of this type of protease [43,52].

## 3. Strategies Applied in Developing Protease Inhibitor

Several strategies have been adopted to develop 3CL^pro^ inhibitors since the outbreak of COVID-19 [30]. These strategies include drug repurposing, structure-based drug design, and fragment-based drug design. The effect of the inhibitors of SARS-CoV 3CL^pro^ on viral replication has also been evaluated using cell-based assays. Accumulated studies have furnished some promising inhibitors for further development.

### 3.1. Drug Repurposing

**Figure 3:**
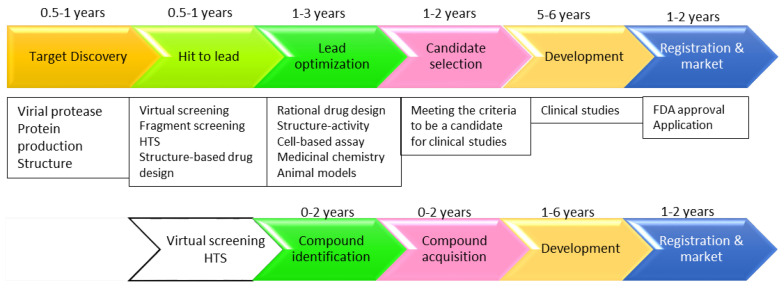
De novo drug discovery versus drug repurposing. The time required from hit identification to lead optimization (**upper panel**) is saved in drug repurposing (**lower panel**). In the case of SARS-CoV-2 3CL^pro^, virtual screening, biochemical, and cell-based assays were applied to identify protease inhibitors from FDA-approved drugs. The duration required in individual processes is based on [59], which gives detailed information for drug repurposing. It is worth mentioning that the timeline for COVID-19 might be different from other diseases due to its pandemic status. HTS, high throughput screening. FDA, the Food and Drug Administration.

Drug repurposing was immediately pursued to explore treatments for COVID-19 [56,57]. Repurposing is also known as repositioning, redirecting, and reprofiling [58]. It is a step to explore the application of an approved drug to treat different diseases from what it was originally developed for. It is well known that a traditional de novo drug discovery takes several years, making drug repurposing a prompt method in the search of medications for SRAS-CoV-2. As approved drugs have gone through clinical studies, important parameters of these drugs, such as pharmacokinetics, pharmacodynamics, and toxicity, have been well characterized (Figure 3). The identified drug can be quickly brought into clinical studies in a short period of time [59]. As the outbreak of COVID-19 caused a worldwide public health emergency, drug repurposing was immediately employed to combat this disease [60,61,62]. Several existing antivirals for SARS-CoV, MERS, malaria, and human immunodeficiency virus (HIV) were tested to treat COVID-19 [63,64,65,66,67], and some drugs have entered into clinical studies [10,63]. The following strategies were applied to identify drugs that can be used to treat COVID-19 by targeting 3CL^pro^.

#### 3.1.1. Virtual Screening

Virtual screenings were performed to identify SARS-CoV-2 3CL^pro^ inhibitors from approved drug libraries [37,68,69,70]. The following strategy was utilized in virtual screening: preparing structural models, selecting drug libraries, specifying a ligand binding site, performing docking, and selecting identified drugs [71,72,73]. In one study, velpatasvir and ledipasvir were selected from a library of 7173 purchasable drugs as supposed inhibitors of SARS-CoV-2 3CL^pro^ [37]. In another virtual screening, four molecules, namely, prulifloxacin, bictegravir, nelfinavir, and tegobuvi, were identified to bind to SARS-CoV-2 3CL^pro^ [68]. In addition, docking and biochemical studies were carried on to repurpose drugs for COVID-19 [74]. One study adopted the crystal structure of SARS-CoV-2 (PDB ID 6LU7) in a docking experiment. Seven identified drugs, namely, pimozide, ebastine, rupintrivir, bepridil, sertaconazole, rimonabant, and oxiconazole, exhibited half maximal inhibitory concentration (IC_50_) values against protease below 100 µM in a biochemical assay [74]. A computer-aided drug discovery protocol was also applied to repurpose approved drugs. This method was named SCAR (steric-clashes alleviating receptors) by which a covalent inhibitor of a target was able to be identified. Eleven potential covalent inhibitors of SARS-CoV-2 3CL^pro^ were identified. These inhibitors might form a covalent bond with Cys145 [75]. Covalent inhibitors have several advantages over noncovalent ones, but no further biochemical or biophysical studies have been conducted to validate the identified drugs [75]. Although these studies are very useful for quickly identifying interesting candidates [76], further biochemical, biophysical, and cell-based identification are essential to confirm activities of these drugs [77].

#### 3.1.2. Artificial Intelligence (AI) Technology

AI technology is able to simulate the human intelligence process using computers [78]. This technology can provide more reliable results as a learning process is taken before the screening is carried out [78]. AI has been applied to screen the approved drug library for potential antivirals against COVID-19 [79,80,81]. Various approved drugs were identified to have potential to inhibit coronavirus after AI learning and prediction processes [82]. A study showed that eight AI-identified drugs were active against viral proliferation of a feline infectious peritonitis virus in a cell-based assay [79]. A similar method will be applied to identify potential inhibitors of SARS-CoV-2 3CL^pro^. A recent study also reported application of AI to design novel inhibitors of SARS-CoV-2 3CL^pro^. An advanced deep Q-learning network with the fragment-based drug design (ADQN-FBDD) was developed to generate lead compounds targeting SARS-CoV-2 3CL^pro^. A total of 47 lead compounds were generated, and their structural information is accessible in a designated library [83]. As AI is able to offer reliable analysis of a potential protease inhibitor, it will play a role in developing SARS-CoV-2 3CL^pro^ inhibitors through drug repurposing or novel drug design [81].

#### 3.1.3. Other Approaches

In addition to computer-based methods applied in drug repurposing, other methods have been employed to identify potential antivirals of SARS-CoV-2 from the FDA-approved drug library. To evaluate the efficacy of a drug, a ferret model for SARS-CoV-2 infection and transmission was established. The efficacy of lopinavir–ritonavir, hydroxychloroquine sulfate, and emtricitabine–tenofovir for SARS-CoV-2 was evaluated using this model and only emtricitabine–tenofovir treatment was able to lower virus titers [84]. Lopinavir–ritonavir is an antiviral that targets the active site of HIV protease [85,86]. Lopinavir–ritonavir was able to reduce the virus load of SARS-CoV, but no benefit was observed in COVID-19 patients in several clinical studies [86,87]. High-throughput screening of an approved drug library using a biochemical assay identified a few potent inhibitors, and several drugs were able to inhibit protease activity [31].

It has been noted that drug repurposing is one approach to identify inhibitors of SARS-CoV-2 3CL^pro^ as some approved drugs exhibited efficacy in clinical studies [88,89]. De novo drug design is still needed to develop protease inhibitors as no approved SARS-CoV-2 3CL^pro^ inhibitor is available. In addition, inhibitors of SARS-CoV 3CL^pro^ could serve as a starting point for developing more potent inhibitors against both SARS-CoV-2 3CL^pro^ and other coronavirus proteases. Several review articles have summarized the application of drug repurposing to combat SARS-CoV-2 through other mechanisms [90,91,92,93,94,95].

### 3.2. Computer-Aided Inhibitor Design

Structure-based in silico virtual drug screenings have been applied to identify SARS-CoV 3CL^pro^ inhibitors [96,97]. As some plants exhibit antiviral activities, virtual screenings were performed to identify protease inhibitors from compounds extracted from these plants [98]. Using a docking method, andrographolide from *Andrographis paniculata*, which has exhibited antiviral activity against some viruses, was docked into the active site of SARS-CoV-2 3CL^pro^ [99]. Screening was also carried out to identify protease binders from a medicinal plant library containing 32,297 potential antiviral phytochemicals [100]. Ten hits were identified to possess potential anti- SARS-CoV-2 activities. These hits might serve as lead molecules for drug development. Virtual screening was also pursued to identify hits from other compound libraries [73]. One study applied a deep docking strategy to analyze a compound library composed of 1.3 billion compounds. Approximately 1000 compounds were identified as potential protease binders [101]. These compounds are publicly available, which is very helpful for researchers to obtain detailed information. These identified compounds might be important for further drug development [101]. Molecular interactions between SARS-CoV-2 3CL^pro^ and natural compounds were also analyzed through docking and molecular dynamics (MD) simulation. Amentoflavone and puerarin were considered to be potential protease inhibitors [102]. In addition, computer-aided method can be adopted to design inhibitors, and several small-molecule compounds have been designed [103,104]. An advantage of this type of screening is that a large number of compounds can be analyzed rapidly, although biophysical and biochemical assays are required to confirm the activity and protease binding of the identified compounds.

### 3.3. Peptidic Inhibitors/Peptidomimetics

**Figure 4:**
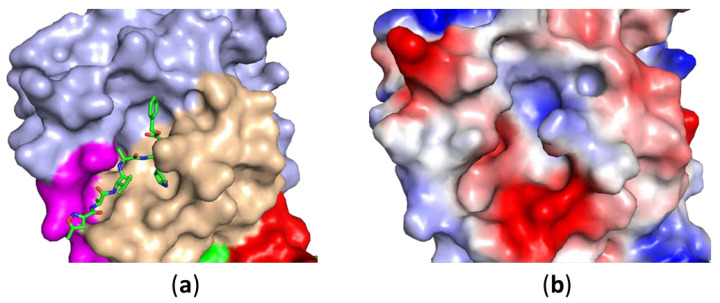
The substrate binding site of SARS-CoV-2 3CL^pro^. (**a**) A cocrystal structure of SARS-CoV-2 3CL^pro^ with an inhibitor (N3) is shown. (**b**) Surface charge analysis of the active site of the protease. The structure (PDB ID 6LU7) is shown using PyMOL (https://pymol.org/2/). The protease in the absence (**a**) and presence (**b**) is shown in the same orientation. The inhibitor is shown as green sticks. Only domains I and II of one protomer of the protease is shown for clarity.

3CL^pro^ binds to a peptide sequence, and peptidic inhibitors derived from its substrate have been developed against SARS-CoV 3CL^pro^ [105,106]. SARS-CoV-2 3CL^pro^ has a substrate pocket located at 3C protease-like domains I and II (Figure 4) [36]. To convert a substrate into an inhibitor, the following approaches can be applied [107]. Firstly, amino acids of the substrate can be mutated to improve the binding affinity to the protease. Secondly, the length of the substrate can be varied to increase its potency to protease activity. Lastly, a warhead can be attached to a peptide to improve the potency [108]. Quite a few warheads, such as aldehydes, Michael acceptors, and epoxy ketones, have been utilized in peptidic inhibitors [106,109]. A peptidic inhibitor with a warhead is able to form a covalent bond with the protease through reaction with the side chain of cysteine or serine in other proteases. Peptidic inhibitors have to be modified into drug-like molecules through peptidomimetics, which can be achieved by reducing the molecular weight, modifying the side chains of amino acids, altering the backbone, or other strategies to improve stability of the peptide [110]. One of the advantages of peptidic inhibitors is that they might have broad-spectrum activity against similar proteases [111,112]. One disadvantage is that it might be challenging to convert a peptidic inhibitor into a drug-like molecule as slight modifications on a side chain of a residue might abolish the activity of the inhibitor [113,114].

**Figure 5:**
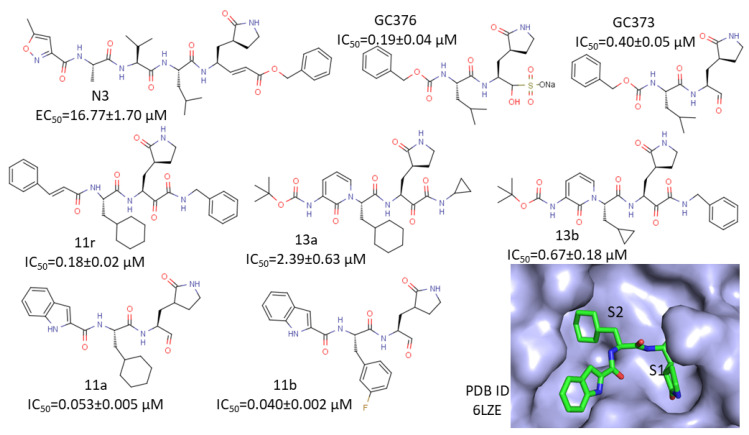
Some peptidic inhibitors of SARS-CoV-2. Structures and their half maximal inhibitory concentration values/ half-maximal effective concentration (IC_50_s/EC_50_s) against SARS-CoV-2 3CL^pro^ are shown. The binding site 11a with SARS-CoV-2 3CL^pro^ is shown. The inhibitor 11a is shown as sticks, and the protease is shown as a surface. S1 and S2 indicate the binding sites for P1 and P2 residues of the inhibitor.

The broad-spectrum inhibitor N3 (Figure 5) is a peptidic inhibitor developed from computer-aided design [115], which was able to inhibit 3CL^pro^ from SARS-CoV and MERS-CoV [116,117]. N3 is indicated to be an irreversible inhibitor of SARS-CoV-2 3CL^pro^. N3 inhibits the protease activity through two steps: protease binding and formation of covalent bond. The inhibitor is first bound to the protease active site to bring a warhead to a close proximity to Cys145. A covalent bond is then formed with the cysteine residue to suppress the protease activity. N3 exhibited a very strong inhibition of SARS-CoV-2 3CL^pro^ with a half-maximal effective concentration (EC_50_) value of 16.77 µM in a cell-based assay. The crystal structure of SARS-CoV-2 3CL^pro^ in a complex with N3 confirmed the mode of action of this inhibitor. The Michael addition was observed, and the P3 might tolerate modifications as the side chain of Val was water-exposed [54].

Rational drug design of peptidic inhibitors was performed for a series of α-ketoamides, which are broad-spectra of 3CL^pro^ of several viruses [36]. The starting inhibitor (11r, Figure 5) exhibited an EC_50_ value of 400 picomolar against MERS in a cell-based assay using Huh7 cell line. This inhibitor exhibited micromolar EC_50_ values against SARS-CoV and a few other viruses in a cell-based assay. Modifications of the inhibitor resulted in 13a with an improved half-life in plasma and increased its solubility to weaken binding to plasma protein (Figure 5), but this compound showed loss of activity against 3CL^pro^ of SARS-CoV-2. Replacing the P2 cyclohexyl moiety with a smaller cyclopropyl resulted in an inhibitor (13b) (Figure 5), which exhibited an IC_50_ values of 0.67 ± 0.18, 0.90 ± 0.29, and 0.58 ± 0.22 µM for proteases of SARS-CoV-2, SARS-CoV, and MERS-CoV, respectively. Absorption–distribution–metabolism–excretion (ADME) properties of 13a and 13b were investigated, and the result indicated that these compounds had great potential to be developed into antivirals [36].

The dipeptide inhibitor GC376 was able to inhibit 3CL^pro^ of MERS [118]. A recent study showed that GC376 (Figure 5) and its analog GC373 were effective inhibitors of SARS-CoV-2 3CL^pro^ [53,119]. An assay was developed using a peptide substrate with an anthranilate–nitrotyrosine donor–acceptor pair in the study. GC376 and GC373 exhibited IC_50_s of 0.19 and 0.40 µM against SARS-CoV-2 3CL^pro^, respectively. These two inhibitors also exhibited nanomolar IC_50_s against SARS-CoV 3CL^pro^. Structures of complexes demonstrated that both compounds were covalently attached to CYS145 of SARS-CoV-2 3CL^pro^ as a hemithioacetal and explained the low IC_50_s of these compounds [53]. Plaque reduction assays were performed using infected Vero E6 cells to evaluate cellular activities of GC376 and GC373. EC_50_ values for GC373 and GC376 were 1.5 and 0.92 μM, respectively. Other potent peptidic inhibitors were developed with a similar warhead. One inhibitor (11a) containing a cyclohexyl moiety and an indole group at the P3 position exhibited (Figure 5) an IC_50_ of 0.05 ± 0.005 µM against SARS-CoV-2 3CL^pro^ [31]. Another compound (11b) with a 3-fluorophenyl group at the P2 position exhibited similar IC_50_ against SARS-CoV-2 3CL^pro^ (Figure 5). Toxicity studies suggest that compound 11b is a promising candidate for clinical trials [31]. Some α-ketoamide inhibitors are able to efficiently suppress protease activity (Figure 5) [36]. Taken together, quite a few peptidic inhibitors exhibited a potency to inhibit the protease activity of 3CL^pro^ of SARS-CoV-2 and might be candidates for clinical studies. Although these inhibitors are able to suppress the protease activity, further optimizations might be required due to the challenges of peptidic inhibitors [113].

### 3.4. High-Throughput Screening

**Figure 6:**
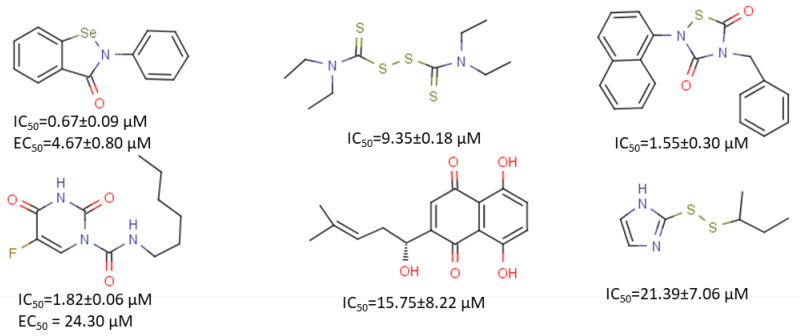
Compounds identified through high-throughput screening. Structures, half maximal inhibitory concentration values/ half-maximal effective concentration (IC_50_s/EC_50_s) (when applicable) of compounds are shown. Please refer to [54] for more details.

A high-throughput screening of a library of 50,000 drug-like small molecules was carried out using a quenched fluorescence resonance energy transfer assay to identify SARS-CoV 3Cl^pro^ inhibitor. A total of 572 hits were identified in primary assay, and five novel small molecules were confirmed using a biochemical assay [120]. These hits exhibited IC_50_ values of 0.5–7 μM against SARS-CoV 3CL^pro^. A similar study was conducted to identify inhibitors of SARS-CoV-2 3CL^pro^. A high-throughput screening of a library of approximately 10,000 compounds was performed, which identified two clinically approved drugs, namely, disulfiram and carmofur, and five drug candidates, namely, ebselen, shikonin, tideglusib, PX-12, and TDZD-8) [54]. These compounds exhibited measurable IC_50_ against SARS-CoV-2 3CL^pro^ (Figure 6). Three compounds, namely, ebselen, PX-12, and carmofur, formed a covalent bond with Cys145 of SARS-CoV-2 3CL^pro^, which was confirmed by mass spectrometry. The cocrystal structure of SARS-CoV-2 3CL^pro^ with carmofur was determined, showing that hydrophobic tails occupied S2 subsite of the protease [55]. Carmofur exhibited an EC_50_ of 24.30 μM in a cell-based assay, and it might be a good candidate to replace the Leu residue at the P2 position of a peptidic inhibitor. Although not all the identified compounds exhibited antiviral activity in a cell-based assay, these hits could serve as a starting point for further improvement. Further chemical modification is still needed to improve their potency.

### 3.5. Fragment-Based Drug Design

Fragment-based drug discovery was pursued to develop novel protease inhibitors [121]. Computational methods were applied to identify hits from a fragment library of 191,678 fragments. The identified hits binding to adjacent subpockets were tailored to other known inhibitors to generate novel molecules. The binding between these novel compounds and a protease was confirmed through docking and MD simulation methods. This study provides a novel strategy to design novel compounds that might inhibit SARS-CoV-2 3CL^pro^. Fragment screening using X-ray crystallography was performed as an effort to combat COVID-19, furnishing diverse starting scaffolds for further optimization. Diamond (UK) was able to produce recombinant SARS-CoV-2 3CL^pro^ based on a recent publication [31]. The structure of the free protease indicates that the active site of the protease is solvent exposable and suitable for fragment screening. As of April 2020, more than 1500 crystals were obtained and analyzed, which resulted in the identification of 71 fragments binding to the active site of SARS-CoV-2 3CL^pro^. A total of 48 fragments were shown to bind covalently to the protease. Experimental details, structures of fragments, and costructures of protease with these fragments are available for researchers (https://www.diamond.ac.uk/covid-19/for-scientists/Main-protease-structure-and-XChem.html). Fragment growth can be carried out according to the structural information. This work will surely benefit the drug discovery effort to combat COVID-19 by offering high-quality data and novel fragments, which saves tremendous amount of time and energy in fragment screening. In addition to identifying fragments by virtual and experimental approaches, fragments were also generated from a known inhibitor to understand interactions between a protease and the ligand. The peptidic inhibitor N3 was segmented into five fragments and the binding energy of fragments with the protease was analyzed. This study identified important residues for inhibitor binding, which provides useful information for rational drug design [122]. Fragments might not exhibit any activity to suppress the protease activity due to their low binding affinity to the protease. With the structural information, further fragment growth can be carried out to develop small-molecule protease inhibitors.

### 3.6. Other Inhibitors

A structural and evolutional study suggested that it might be challenging to develop small-molecule inhibitors of SARS-CoV 3CL^pro^ [123]. This might be true as there has not been any small-molecule drug that works actively against SARS-CoV 3CL^pro^ since the viral outbreak in 2003. Allosteric inhibitors might have a high probability against this protease. A recent docking study identified some FDA-approved drugs that were bound to an allosteric site of SARS-CoV 3CL^pro^ [124]. Further experiments are required to prove modes of action for the identified compounds. It is challenging to develop this type of inhibitors as suitable assays are critical in developing allosteric inhibitors. Another strategy to inhibit protease activity is to disrupt dimer formation by preventing molecular interactions of the N-finger and domain II. A study on 3CL^pro^ of SARS-CoV demonstrated that an octapeptide derived from the N-finger of the protease was able to disrupt protease dimerization [125]. Therefore, a potent peptidic inhibitor might be developed, although more work is still needed to prove the feasibility.

## 4. Perspectives

The outbreak of COVID-19 has caused a health—and even a life—threat to the global population and seriously affected the living styles of most people. In the fight against SARS-CoV-2, data analysis has been playing an important role in understanding, managing, and giving insights into developing strategies to prevent the spread of the virus [126]. Researchers have designed some inhibitors of SARS-CoV-2 3CL^pro^ for developing antivirals. The following aspects are critical and play an important role in developing novel and potent protease inhibitors.

Data sharing is helpful for researchers to speed up their studies. Many research articles are available online without a peer review. These articles might require some revisions, but they provide fast and useful information to understand this disease on time. In addition, the availability of viral genome sequence, identified FDA-approved drugs, and screened hits using different methods have made it possible for researchers to choose suitable strategies to conduct corresponding studies right after the outbreak of COVID-19.

Drug repurposing has been applied to quickly seek possible medicines for the virus. Although the success might not be guaranteed due to many reasons, this method is highly recognized by scientists. It has become a routine method in drug discovery, especially for the virus causing such a pandemic. When the structure of a target is known, docking through a suitable program can identify potential compounds binding to the target. Suitable biochemical, biophysical, and other assays are required to validate the hits. Modification of the identified drug might be needed to improve its potency.

Peptidic inhibitors are of great interest in the development of protease inhibitors. Several peptidic inhibitors have exhibited potent activities against SARS-CoV-2 3CL^pro^. These inhibitors will be further evaluated using animal models as different cell-based assays might give various results. Improving ADME properties are critical for this type of inhibitors. Accumulated studies suggest that it is feasible to develop peptidic antivirals.

Structure-based drug design is key to antiviral development. The available cocrystal structures of SARS-CoV-2 3CL^pro^ with different types of inhibitors are critical for improving the potency and understanding the mode of action of inhibitors. This approach is also important in fragment-based drug design by providing efficient strategies for fragment growth. Quite a few small-molecules have been identified through docking and high-throughput screening. Medicinal chemists are important for modifying these compounds to improve their potency. Cocrystal structures of proteases with these compounds are very helpful for guiding compound optimization.

Compounds inhibiting protease activity through other mechanisms, such as allosteric binding and disrupting dimeric structure of the protease, might be possible, although a suitable assay is required. No allosteric inhibitor of SARS-CoV-2 3CL^pro^ has been identified so far. Despite the challenges in novel drug discovery, accumulated studies shed light on the development of antivirals by targeting 3CL^pro^.

## Figures and Tables

**Table 1:** Some crystal structures of 3CL^pro^ of SARS-CoV-2 ^1^.

PDB ID	Remarks	Reference
6Y2G	Complex structure	[36]
6Y2F	Complex structure	[36]
6Y2E	Free protease	[36]
6LZE	In complex with 11a	[31]
6M0K	In complex with 11b	[31]
6WTJ	In complex with GC376	[53]
6WTK	In complex GC373	[53]
7BQY	In complex with N3	[54]
6LU7	In complex with N3	[54]
7BUY	In complex with carmofur	[55]

^1^ Over 100 structures are deposited in the protein data bank (PDB), and only structures associated with publications are shown in the table.
